# Isolation and characterization of malaria PfHRP2 specific V_NAR_ antibody fragments from immunized shark phage display library

**DOI:** 10.1186/s12936-018-2531-y

**Published:** 2018-10-24

**Authors:** Chiuan Herng Leow, Katja Fischer, Chiuan Yee Leow, Katleen Braet, Qin Cheng, James McCarthy

**Affiliations:** 1 QIMR Berghofer Medical Research Institute, Brisbane, Australia; 2 School of Medicine, University of Queensland, Brisbane, Australia; 3 Institute for Research in Molecular Medicine (INFORMM), Universiti Sains Malaysia, Penang, Malaysia; 4 Australian Army Malaria Institute, Brisbane, Australia; 5 Department of Research, BioMARIC, Zwijnaarde, Belgium

## Abstract

**Background:**

Malaria rapid diagnostic tests (RDTs) represent an important antibody based immunoassay platform. Unfortunately, conventional monoclonal antibodies are subject to degradation shortening shelf lives of RDTs. The variable region of the receptor (V_NAR_) from shark has a potential as alternative to monoclonal antibodies in RDTs due to high thermal stability.

**Methods:**

In this study, new binders derived from shark V_NAR_ domains library were investigated. Following immunization of a wobbegong shark (*Orectolobus ornatus*) with three recombinant malaria biomarker proteins (PfHRP2, PfpLDH and Pvaldolase), a single domain antibody (sdAb) library was constructed from splenocytes. Target-specific V_NAR_ phage were isolated by panning. One specific clone was selected for expression in *Escherichia coli* expression system, and study of binding reactivity undertaken.

**Results:**

The primary V_NAR_ domain library possessed a titre of 1.16 × 10^6^ pfu/mL. DNA sequence analysis showed 82.5% of isolated fragments appearing to contain an in-frame sequence. After multiple rounds of biopanning, a highly dominant clone specific to PfHRP2 was identified and selected for protein production in an *E. coli* expression system. Biological characterization showed the recombinant protein expressed in periplasmic has better detection sensitivity than that of cytoplasmic proteins. Assays of binding activity indicated that its reactivity was inferior to the positive control mAb C1–13.

**Conclusions:**

Target-specific bacteriophage V_NAR_s were successfully isolated after a series of immunization, demonstrating that phage display technology is a useful tool for selection of antigen binders. Generation of new binding reagents such as V_NAR_ antibodies that specifically recognize the malaria biomarkers represents an appealing approach to improve the performance of RDTs.

**Electronic supplementary material:**

The online version of this article (10.1186/s12936-018-2531-y) contains supplementary material, which is available to authorized users.

## Background

Malaria remains one of the most life-threatening infectious diseases in the world. Five species of *Plasmodium* cause malaria in humans. Of these species, infection with *Plasmodium falciparum* is the most prevalent and lethal, causing significant morbidity and mortality worldwide [1]. Most of the *P. falciparum*-detecting rapid diagnostic tests (RDTs) target histidine-rich protein 2 (PfHRP2) [2]. PfHRP2 is a water-soluble protein that is produced by all asexual stages of *P. falciparum* including gametocytes. This protein is abundantly expressed in the red cell, released during rupture of infected red cells and can remain in the blood for up to 28 days after the initiation of anti-malarial therapy, making it an excellent biomarker for diagnosing *P. falciparum* infections [3]. *Plasmodium* lactate dehydrogenase (pLDH), and fructose 1,6-biphosphate aldolase (Aldolase) are biomarkers commonly used for the detection of non-*P. falciparum* human malaria infections (species specific or PAN specific) and *P. vivax* infections, respectively [4, 5].

Unfortunately, the degradation of sensitive capture and detecting antibody reagents in malaria RDTs [6] can shorten the shelf lives of RDTs and may also result in false negative diagnosis and eventually delay the treatment time if undetected [7]. Antibodies with better stability profiles would improve the stability of RDTs. However, despite early attempts to engineer antibodies into more robust antibody fragments [8, 9], separating the VH and VL domains while retaining antibody specificity has proven to be difficult [10, 11]. In nature, sharks are the most ancient phylogenetic vertebrate group possessing the complete molecular components of an adaptive immune system [12, 13]. In contrast to immunoglobulin (Ig) isotypes in higher mammals, the immunoglobulin new receptor (IgNAR) of sharks are unusual antibodies that lack light chains and, therefore, exist as homodimers of a heavy chain [14]. Immune electron microscopy indicated that the IgNAR heavy chain contains one variable domain of (V_NAR_) and five constant (C) domains [15]. Similar to V_HH_ in the camelid family, V_NAR_ domains can function as soluble single domains which are capable of antigen binding [15]. These single domain fragments display excellent solubility and high thermostability due to substitutions of amino acids at VH–VL interaction, making the interface more hydrophilic compared to the hydrophobic interface present in conventional antibodies [14, 16].

Similar to the variable domains of conventional immunoglobulin scaffolds, shark V_NAR_ have been determined to have four highly conserved framework regions (FR) and three highly variable complementary determining regions (CDRs). The deletion of a large portion of FR2–CDR2 has therefore made V_NAR_ the smallest variable domain, with size of ~ 12 kDa [14]. In addition, shark V_NAR_ domains possess an extraordinary CDR3 domain which is much longer than that of conventional antibodies. Therefore, the penetration capability of V_NAR_ is perceived much easier to reach to the cleft region of the target antigen [17, 18]. Thus far, V_NAR_ is recognized as the smallest natural single domain antibodies (sdAbs) found to date in the animal kingdom [14, 19].

The selection of a suitable expression system is vital to ensuring the solubility and correct folding required for expression of functional V_NAR_ protein. Bacterial expression systems are often the first choice in the laboratories for the production of recombinant proteins for therapeutic and diagnostic applications [20–22]. It is amenable to produce recombinant proteins for a range of biological applications [23].

Due to the high numbers of cysteine residues, the soluble expression of V_NAR_ constructs in bacterial hosts has been extensively studied by a range of laboratories [24, 25]. Thus far, V_NAR_ proteins have been reportedly expressed in bacteria system using proprietary expression vectors such as pIMS100 [25], pIMS147 [26], Pecan22 [27, 28] pAK-300 [29], and pET-28a [30]. In addition, the PelB leader peptide has been commonly used as a fusion protein partner for bacterial periplasmic targeting [31, 32].

In the present work, new antigen binders against malaria biomarkers were investigated by constructing an immune shark V_NAR_ domains library, selecting high affinity binders against malaria antigens by biopanning, followed by protein expression in various *Escherichia coli* expression systems. The binding ability of recombinant proteins to target antigens was then compared with a commercial mouse mAb.

## Methods

### Antigens and adjuvants

Recombinant malaria proteins rPfLDH (6 mg/mL), and rPvAldolase (1.6 mg/mL) were purchased from Standard Diagnostic Inc, Korea. Recombinant protein rPfHRP2 (5 mg/mL) was produced by Lee [33]. All three proteins were diluted in PBS to a final concentration of 250 µg/mL. Each diluted protein was then aliquoted (1 mL) in a fresh 1.5 mL tube and kept at − 20 °C until use. Complete Freund’s adjuvant (CFA) and incomplete Freund’s adjuvant (IFA) were used for shark immunization. CFA was only used during the first immunization and replaced by IFA during subsequent immune boosting of a wobbegong shark. Both CFA and IFA were purchased from Sigma-Aldrich (USA) and were kept at 4 °C until use.

### Immunization of a wobbegong shark

All animal handling work was approved by Animal Ethics Committees of UQ and QIMR Berghofer MRI. A banded wobbegong shark (*Orectolobus ornatus*) weighing 2.1 kg, was housed at the Moreton Bay Research Station, University of Queensland. The three malaria recombinant soluble proteins (rPfHRP2, rPfLDH and rPvAldolase at 250 µg/mL each) were emulsified in equal amounts of CFA and subcutaneously injected in the pectoral fin as a mixed-antigens cocktail. Subsequent boosts were administered intravenously in the caudal vein. To enable production of the immune library, the wobbegong shark was given an initial injection with 2 mL mixed-antigen cocktail into its lateral fin. For subsequent boosts, 1 mL PBS containing 250 µg/mL of antigens cocktail was mixed with 200 µL IFA and was then injected into the caudal vein intravenously at 4-week intervals. To measure the humoral response of shark IgNAR against malaria antigens, 3 mL blood sample was drawn from the caudal vein. Serum was isolated to determine the reactivity of shark IgNAR plasma against malaria biomarkers by ELISA using a panel of IgNAR-specific monoclonal antibodies (kindly provided by Prof. Flajnik, University of Maryland, Baltimore). When sera titres rose to an appropriate level, the shark spleen and peripheral blood lymphocytes (PBL) were isolated for construction of the immune V_NAR_ library.

### Total RNA extraction and first strand cDNA synthesis

Total RNA extracted from shark spleen and peripheral blood lymphocytes (PBL) was pooled and approximately 20 ng/µL of purified mRNA extracted by Poly(A) Purist Kit (Ambion^®^ Invitrogen, USA) was used as template for first strand cDNA synthesis with addition of Leader-F primer and C-domain R primer (Table 1). The pre-denaturation was set at 94 °C for 2 min. The PCR amplification conditions were set at 35 cycles of 30 s at 94 °C, 30 s at 55 °C, 1 min at 72 °C, and a final extension was at 72 °C for 10 min. The resultant amplicons were separated on an agarose gel and the target band was excised and purified for library construction.

**Table 1:** Primers used for shark V_NAR_ library construction and sequencing

Primers description	Nucleotide sequence (5′→3′)	Applications
Leader-F	ATGAATATTTTCTTGCYGTCAGTCC	1st PCR
C-DomainR	CCTCTCTGTTCTTCRGTTGCAGAGT	1st PCR
FR1F1	RCAWGGGTRGACCAAACACC	Nested PCR
FR4R	TTTCACGGTYARTRCGGTGCC	Nested PCR
T7SelectUP (For)	GGAGCTGTCGTATTCCAGTC	T7 Phage sequencing
T7SelectDOWN (Rev)	AACCCCTCAAGACCCGTTTA	T7 Phage sequencing
T7Seq_For	TAATACGACTCACTATAGGG	pET vector sequencing forward
T7Seq_Rev	CTAGTTATTGCTCAGCGGTG	pET vector sequencing reverse

Association (k_a_), dissociation rates (k_d_), and equilibrium constants (K_D_) of mAbs D2, F9, and C1–13 against rPfHRP2. The analysis for mAbs D2 and F9 were undertaken using the Octet Red platform, whereas mAb C1–13 was determined by BIAcore

### Construction of shark V_NAR_ cDNA library

The purified PCR product was then used as a template for second PCR using nested primers set (FR1F1 and FR4R) shown in Table 1. The nested PCR products (V_NAR_ cDNA) were ligated to *Eco*RI and *Hin*dIII linkers according to manufacturer’s protocol described in OrientExpress™ cDNA Cloning kit (Novagen, EMD Millipore Chemicals, USA). After treating with restriction enzymes *Eco*RI/*Hin*dIII, the digested product (100 µL) were purified by size fractionation through a mini column filled with gel filtration resin. The purified V_NAR_ cDNA was subsequently ligated into *Eco*RI/*Hin*dIII digested T7Select^®^1-1b vector. For the ligation reaction, a 10 µL reaction mixture was set up according to manufacturer’s protocols. On the next day, in vitro Packaging of T7 Bacteriophage was performed by adding 5 µL of ligated V_NAR_/T7Select^®^1-1b product into 25 µL of Packaging Extract. After incubating at room temperature for 2 h, the packaging reaction was terminated by adding 270 µL sterile LB medium and 20 µL chloroform. The mixture was mixed well by inversion prior to undertaking the plaque assay.

### T7 phage biopanning

Three immunotubes (Nunc) coated with 1 mL of recombinant rPfHRP2, rPfLDH and rPvAldolase with respective concentration of 100 µg/mL (Round 1), 50 µg/mL (Round 2), 25 µg/mL (Round 3) and 12.5 µg/mL (Round 4) were incubated overnight at 4 °C on a rotator. After washing and blocking, 1 mL of amplified T7 phage display library (1.5 × 10^11^ pfu/mL) was added to each immunotube and incubated for 1 h at room temperature in a rotator. The unbound phage was removed by washing 5 times with 0.1% PBST (PBS containing 0.1% Tween-20) in round one. The number of washes was increased to 10 times for subsequent rounds of panning. Bound phage were eluted with 1 mL of T7 elution buffer (1% SDS in TBS) of which 250 µL of eluted phage was used to infect host cell BLT5615 (OD 1.0 induced with 1 mM IPTG) for amplification. To enrich specific clones, the amplified phage was subjected to another three rounds of panning as described above. To monitor enrichment, the recovery rate of each round of panning was determined by plaque assay. The targeted T7 phage was scraped and dispersed in 300 µL of phage extraction buffer at 4 °C.

### Determination of V_NAR_ inserts with expected size and in-frame sequence

To determine the DNA sequence of V_NAR_ insert in T7 phage, 2 µL of plaque lysate was used as template for PCR amplification with addition of T7SelectUP (For) and T7SelectDOWN (Rev) sequencing primers. When the amplification was completed, 10 µL of PCR reactions were mixed with 1 µL of 10× loading dye and separated by 1% agarose gel containing 0.5 µL/mL ethidium bromide. V_NAR_ lengths were then determined visually from the agarose gel. PCR product were sequenced as described above. The deduced amino acids of V_NAR_ were aligned using ClustalW (http://www.ebi.ac.uk/Tools/msa/clustalw2/) and the plaque containing intact V_NAR_ region was determined using BLAST.

### Expression of His6-tagged H8VNAR protein

Recombinant V_NAR_ proteins were expressed in bacterial cytoplasmic and periplasmic compartment. Briefly, the selected V_NAR_ gene fragment H8VNAR was cloned into the expression vectors pET-28a (cytoplasmic expression) and pDSB-28Y (periplasmic expression) prior to transformation in *E. coli* BL21(DE3) competent cells. After sequence confirmation of the constructs, the expression of His-tagged H8VNAR protein was initiated by inoculating 100 µL glycerol stock of transformed BL21 cells in 1 L of fresh 2YT medium containing 30 µg/mL kanamycin and incubating at 37 °C with 200 rpm shaking overnight. The cultures were then induced by IPTG at a final concentration of 0.1 mM, followed by an additional incubation at 20 °C, with shaking at 200 rpm for 16 h.

### Extraction of protein fractions

Overnight cultures were centrifuged at 6000×*g* at 4 °C in an Ultra Centrifuge (Beckman, USA) for 40 min. To extract the soluble protein expressed in cytoplasmic compartment, pelleted bacteria were resuspended in 30 mL cold Tris–phosphate buffer and lysed by passing through a French press. To extract periplasmic proteins, pelleted bacteria were subjected to an osmotic shock. Briefly, pelleted bacteria were resuspended in 30 mL of ice-cold TES buffer (0.2 M Tris–HCl pH 8.0, 0.5 mM EDTA, 20% sucrose). The cell suspension was incubated on ice for 30 min. Cells were lysed by adding 30 mL of 5 mM MgSO_4_ to the cell suspension and further incubation for 30 min on ice. The slurry was then centrifuged at 20,000 rpm for 40 min at 4 °C. The resulting supernatant was collected and twice filtered through 0.45 µm filter. All supernatants collected were then dialyzed overnight against protein binding buffer (50 mM Tris–HCl pH 8.0, 3 mM MgCl_2_, 300 mM NaCl) at 4 °C. The purification of recombinant His6-tagged H8VNAR protein was done using a Nickel-NTA Agarose Resin (Sigma-Aldrich, USA), according to the manufacturer instructions.

### rPfHRP2-specific V_NAR_ protein sandwich ELISA

The purified H8VNAR proteins were analysed by SDS-PAGE (15%) and Coomassie staining. The concentration of protein was quantified by Bradford method [34] using the Bio-Rad Protein Assay (Bio-Rad Laboratories) according to manufacturer instructions. To determine the binding efficacy of recombinant antibodies, H8VNAR proteins (1 µg/mL or ~ 70 nM) purified from both cytoplasmic and periplasmic spaces were used as capture antibodies for the anti-rPfHRP2 assays in a sandwich ELISA. Tenfold serial diluted recombinant PfHRP2 proteins, with starting concentration at 10 µg/mL, were added to each H8VNAR. The commercial mouse mAb C1–13 (10 µg/mL or ~ 70 nM) (kindly provided by Dr. Martin Bubb, National Bioproducts Institute, South Africa) was used to detect captured antigens. The sandwich was detected by goat anti-mouse antibody conjugated to horseradish peroxidase (HRP) (Jackson, USA). Absorbance was read at 405 nm.

### Dot blot analysis

A sandwich format of Dot Blot assay was used to determine the sensitivity of H8VNAR proteins against recombinant PfHRP2 protein. Briefly, H8VNAR proteins diluted in PBS (~ 70 nM) were transferred onto a pre-wetted nitrocellulose membrane (Hybond-C, Amersham Bioscience, UK) using Dot blotter (BioRad, USA). A twofold serial dilution of recombinant PfHRP2 protein was added to the H8VNAR proteins. The nitrocellulose membrane was then removed from the blotter and incubated with antibody C1–13 (~ 70 nM) at room temperature for 1 h. Detection of HRP-conjugated secondary antibody was performed using ECL chemiluminescence detection kit (Amersham Bioscience, USA), following the manufacturer’s instructions. The exposed film was developed by Kodak X-omat imager.

## Results

### Immunization of wobbegong shark and IgNAR ELISA screening

**Fig. 1:**
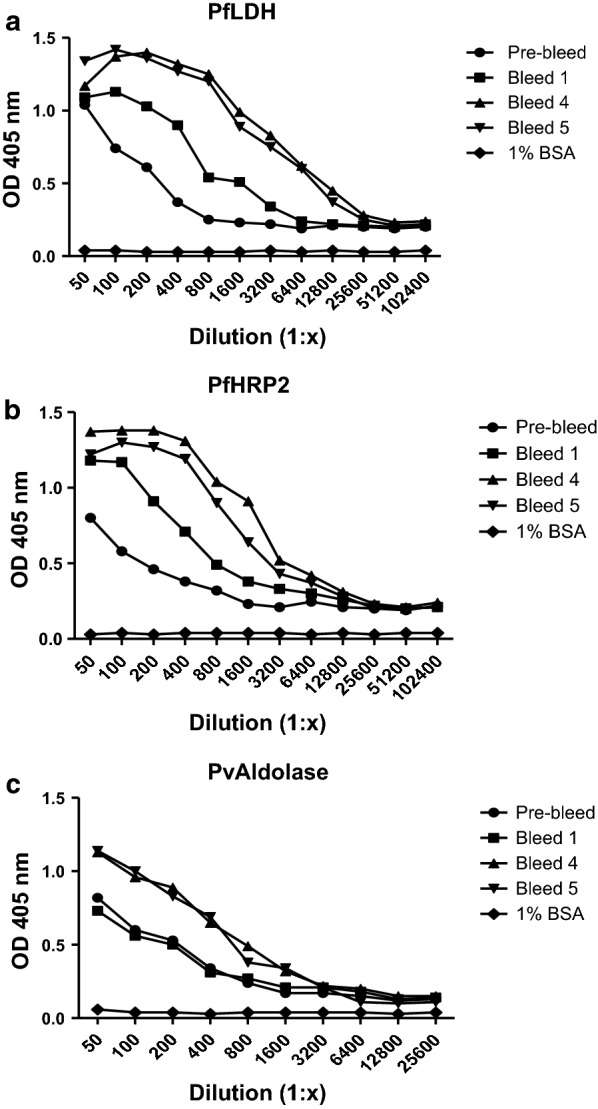
End-titre of shark plasma against three malaria recombinant proteins over course of immunization. Shark plasma reacted against PfLDH (**a**), PfHRP2 (**b**), and PvAldolase (**c**). The baseline antibody response (negative control) was represented by pre-bleed (naïve sera) of wobbegong shark. The immobilized 1% BSA was used as negative antigen and reacted with serial diluted pre-bleed sera

A wobbegong shark was immunized with three malaria antigens, rPfHRP2, rPfLDH, and rPvAldolase. After six immunizations over a 7 months period, the antibody titre specific to the three malaria biomarker had risen compared to the pre-bleed titer, and compared to titters in a naïve (non-immunized) wobbegong as a negative control. As shown in Fig. 1, IgNAR response against all three antigens increased markedly in bleed 4 and 5 compared to pre-bleed and bleed 1. However, antibody levels in bleed 5 were not different to those in bleed 4. The highest shark immune response in bleed 5 was observed against rPfLDH, followed by rPfHRP2, with the antibody titre over 1:51,200 and 1:25,600, respectively. The anti-rPvAldolase IgNAR titre was lower at 1:12,800. As the antibody response was no longer increasing after the fifth boost, the spleen and peripheral blood lymphocytes were harvested from the immunized shark and the total RNA was isolated.

### V_NAR_ domains library construction

An immune IgNAR V region (V_NAR_) phage display library was constructed and screened for V_NARs_ specific to malaria biomarkers. Total RNA was purified from splenocytes and peripheral blood lymphocytes of immunized shark. The successful amplification of V_NAR_ region (nested PCR product) was clearly indicated with a strong band present at 340 bp. After digestion with appropriate restriction enzymes, the DNA was cloned into the T7Select^®^1-1b vector that facilitates expression of phage 10B-V_NAR_ fusion proteins on the phage surface.

### Library characterization

The size of primary library was determined to possess 1.16 × 10^6^ pfu/mL. PCR screening of 132 randomly selected plaques from the primary library revealed that approximately 55% (72/132) of the clones contained inserts of the expected size at (~ 450 bp) (Additional file 1). Nucleotide sequence analysis showed that 82.5% (33/40) of isolated fragments contained an in-frame sequence, while 17.5% (7/40) contained frameshift mutations. Thus, it was estimated that over 80% of the V_NAR_ library with a size of ~ 5.26 × 10^5^ pfu/mL in 50 mL culture encoded functional inserts.

From the V_NAR_ domains library, the amino acid sequences of 33 randomized clones were aligned and directly evaluated by comparison with three V_NAR_ domain control genes derived from *Orectolobus maculates* (wobbegong shark) clones published in the protein database (PDB), namely AY261681 [32], AY261682 [32], and AF466395 [17]. The complete two signature CDR1 and CDR3 regions and four conserved framework regions (FR) were present in each of the sequenced domain of V_NAR_. In addition, the hypervariable (HV) 2 region with shorter length of the amino acid stretch also present. Furthermore, most of the clones demonstrated unique amino acid sequences, with no identical clones detected in the primary library.

### Examination of the quality of V_NAR_ domains library

Significant diversity in the V_NAR_ domains was observed especially in their CDR3 regions. As expected for an adult shark, types 1 and 2 but not type 3 of IgNAR repertoires were represented in the library (Table 2). Considering the number of non-canonical cysteine (Cys) residue(s) encoded within CDR1 and CDR3 regions in each V_NAR_, approximately 88% of the sequenced clones in this library were classified as type 2, whereas 12% were classified as type 1. Generally, type 1 IgNAR V regions have two non-canonical Cys residues in the CDR3, and two additional in FR2 and FR4. Type 2, V regions have two non-canonical Cys residues, one each in CDR1 and CDR3. Unexpectedly, about 27% (9/33) of the clones in this library were found to possess three Cys residues in their CDR3 region.

**Table 2:**
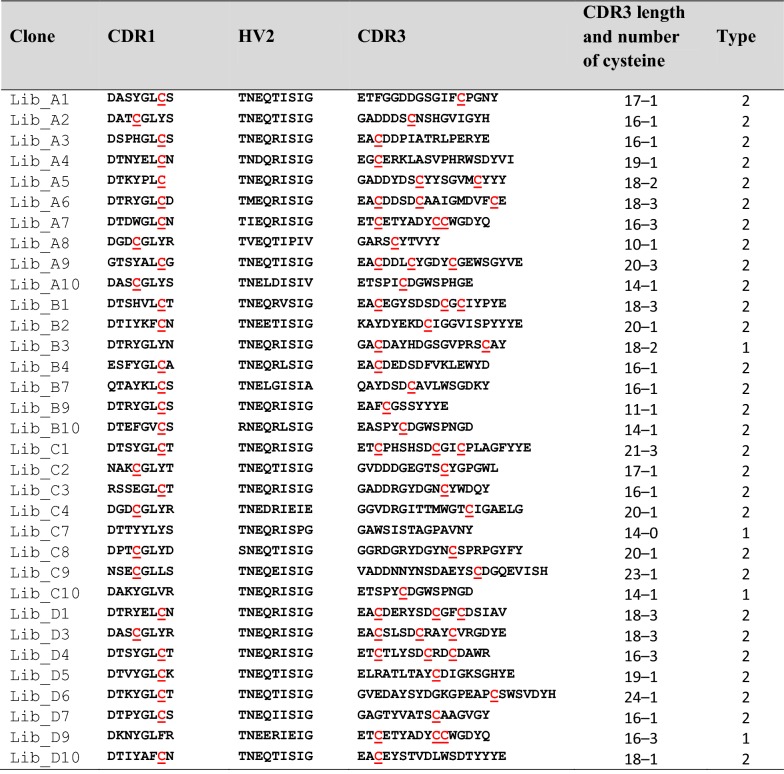
Deduced amino acid sequences in hypervariable regions of V_NAR_ clones randomly selected from primary library

The non-canonical cysteine residue is highlighted in red color. This table also indicates the length of CDR3, number of cysteine residue, and type of IgNAR family for each clone

In terms of the length of CDR3 regions, the encoded amino acid residues ranged from 10 to 24 in residue length, with an average of 17.2 amino acids. Most of the CDR3 regions appeared to generate long loops which predominantly consisted of 14, 16, 18 and 20 amino acid residues. The alignment also showed that a conserved canonical Cys residue at the position 22 was consistently observed in the C-terminal of CDR1 sequence, while mutations were found in the FR regions. Another canonical Cys residue was also detected at the C-terminal of CDR3 regions at position of 84 in all clones studied. The V_NAR_ domains library was evaluated to be of good quality because of: (i) the highly variable CDR3 regions, (ii) the presence of type I and type II of IgNAR family, and (iii) the position of the conserved canonical C residues being similar to the V_NAR_ sequences published in the protein database.

### Biopanning of the immunized V_NAR_ library against three malaria biomarkers

**Fig. 2:**
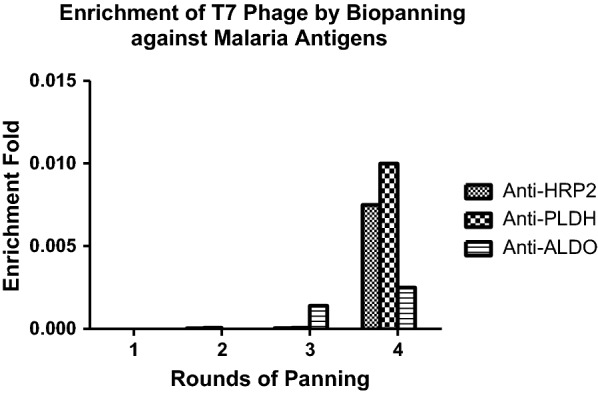
Selection and enrichment of malaria biomarker specific T7 phage after four rounds of biopanning. A plaque assay was performed to measure the size of eluted phage after each round of panning, and the size of amplified phage. The enrichment fold was calculated by dividing the eluted phage by input library size

To screen for target clones, four rounds of panning were performed using an amplified library with the size of 1.5 × 10^11^ pfu/mL. Using this approach, target-specific clones were obtained for each malaria biomarker. The highest phage titre pattern was observed for rPfLDH, followed by rPfHRP2 and rPvAldolase as represented in Fig. 2. Following four rounds of panning, a total of 30 single phage plaques, targeting each antigen were separately picked for further analysis by PCR screening and sequencing. The results indicated that 20 anti-rPfHRP2 clones, 18 anti-rPfLDH clones, and 15 anti-rPvAldolase clones had the expected size and in-frame (ORF) sequences. Only the in-frame clones were chosen for further analysis.

### Competitive biopanning

To avoid cross-reactivity of non-specific clones, a competitive biopanning was applied to further select the strongest unique binders. Briefly, three more rounds of biopanning were carried out by immobilizing the target proteins in immunotubes as described in section T7 Phage Biopanning. Of 53 selected clones isolated from the previous panning, 26 clones with distinct sequence were pooled and subsequently tested for competitive binding to either their putative target ligand or to the other two ligands, to which no binding should occur.

**Fig. 3:**
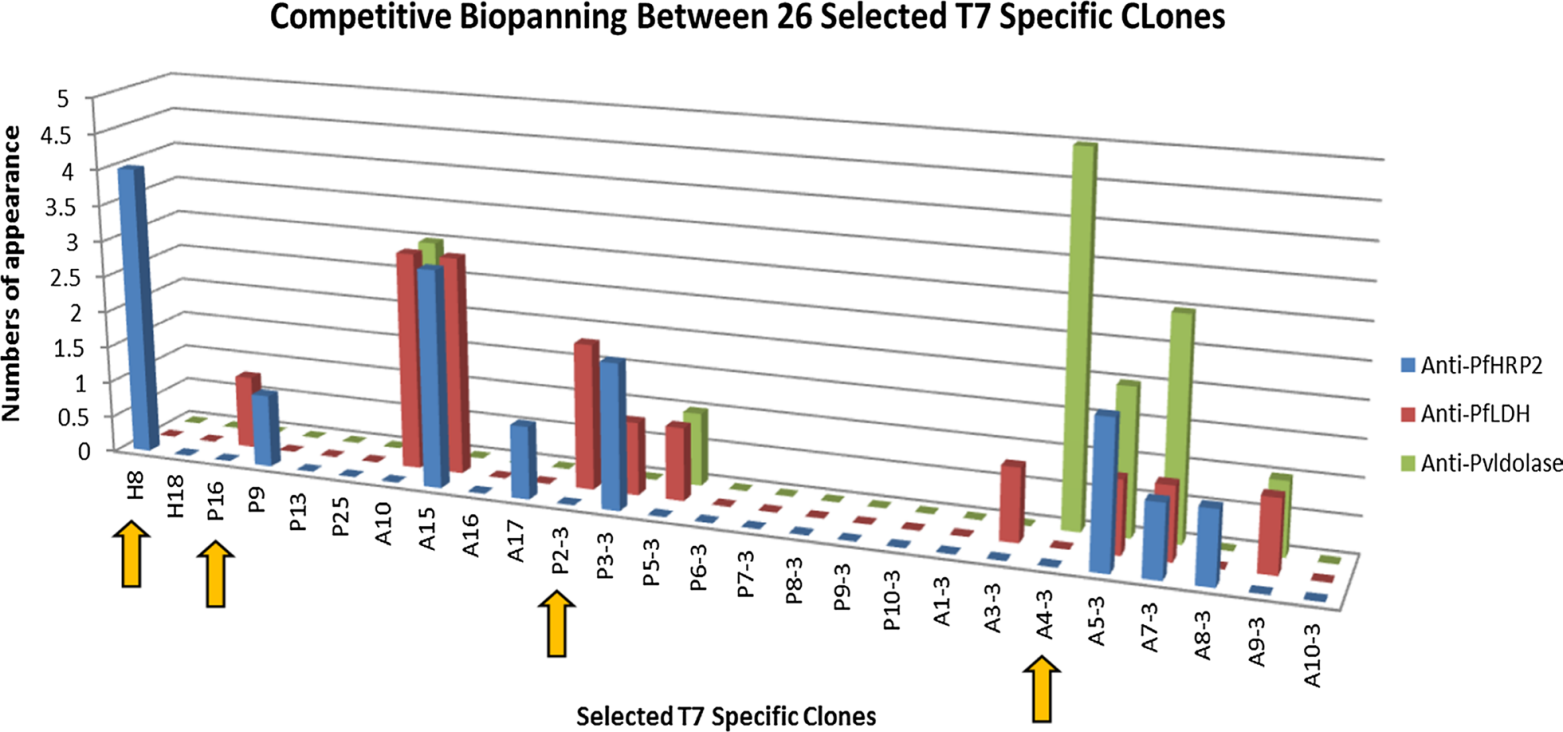
Competitive biopanning between 26 selected T7 specific clones. Twenty-six cloned selected from initial four rounds of biopanning against three different malaria biomarkers were pooled. This pool was tested competitively to select the specific clones, based on high frequency of appearance and absence of cross-reactivity. The colour bars represent the clones isolated from biopanning against different malaria antigen proteins (blue: anti-PfHRP2; red: anti-PfLDH; green: anti-PvAldolase). Yellow arrows point to four clones identified to be specific (H8 as an anti-PfHRP2 clone, P16 and P2–3 as anti-PfLDH clones, and A4–3 as an anti-PvAldolase clone)

Competitive biopanning resulted in 26 clones retained after four rounds of panning. When inspecting the ratio of specific to non-specific binding of the resultant clones, four of the 26 clones were identified to be predominantly reacting to one specific antigen: H8 as an anti-PfHRP2 clone, P16 and P2–3 as anti-PfLDH clones, and A4–3 as an anti-PvAldolase clone (Fig. 3). The clones that showed binding to all antigens such as clones A5–3 and A7–3 were eliminated from further analysis.

### Sequence analysis of targeted clones

**Fig. 4:**
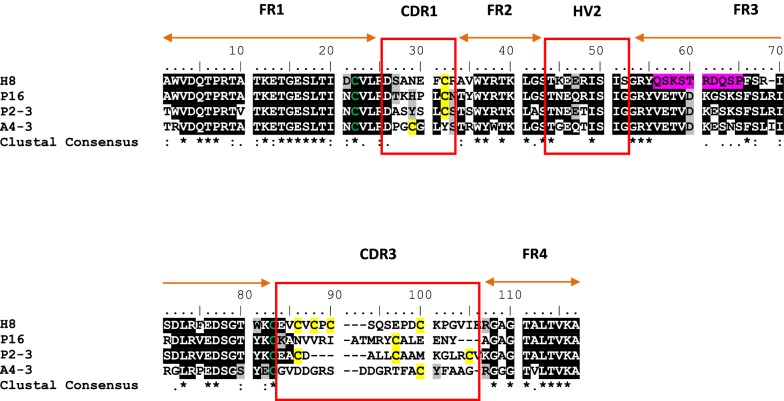
Deduced amino acid residues of four clones isolated after competitive biopanning. The PfHRP2-specific clone was represented by H8; The PfLDH-specific clones were P16 and P2–3; the PvAldolase-specific clone was A4–P4. All FR regions are shown by orange bar. The CDR regions are shown by red box. The canonical cysteine residues in FR1 and FR3 regions are highlighted in green. The non-canonical cysteine residues in CDR1 and CDR3 regions are highlighted in yellow background. The mutation sites in FR3 region of clone H8 is highlighted in pink background

The amino acid sequence of the four final clones isolated following competitive biopanning were aligned (Fig. 4). The clones were found to lack Cys residues in FR2 and FR4 regions. However, inspection of CDR regions revealed that all clones had conventional type 2 IgNAR V regions. No major mutations were identified in FR1, FR2, FR3 and FR4 regions of clones P16, P2–3, and A4–3, except for the FR3 region of H8 clone.

**Fig. 5:**
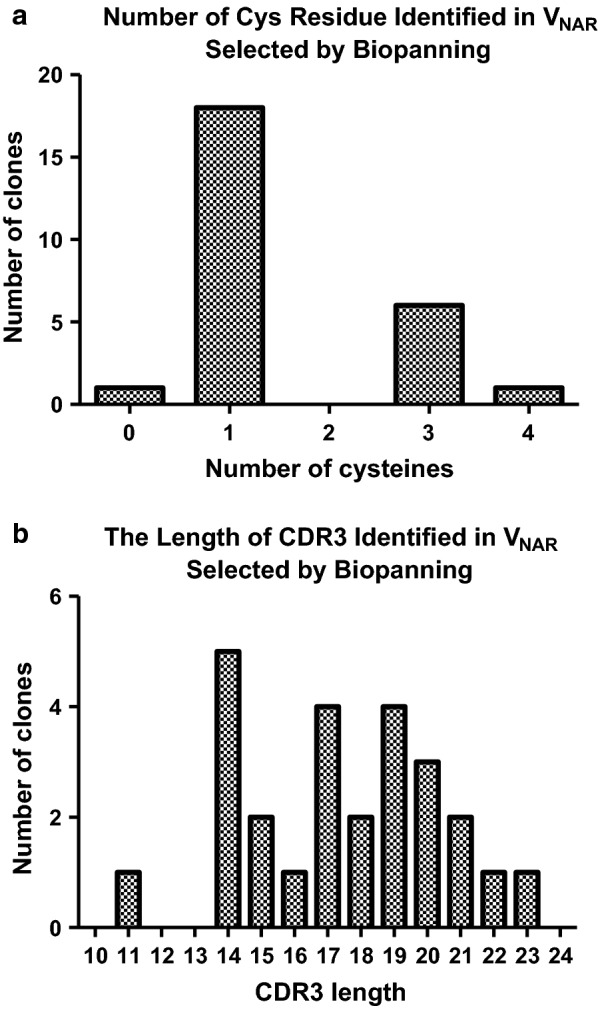
Analysis of deduced amino acids of V_NAR_ clones selected after four rounds of biopanning. **a** The number of cysteine residues and **b** represents the length of the CDR3 region encoded within the V_NAR_ clones

In terms of number of Cys residue, H8 contained four residues in CDR3 region, the largest numbers of Cys residues observed in this library. In comparison, three Cys residues were detected in clone P2–3, and only one in clones P16 and A4–3. All clones were observed to have longer amino acid residues stretches in CDR3 which were more than the average of 17 amino acids of the V_NAR_ library (Fig. 5). The longest CDR3 were H8 and A4–3 with 20 residues, followed by P16 with 19 residues, and P2–3 with 17 residues. Being a highly dominant clone with an average appearance higher than 85% after each round of panning, H8 was selected for recombinant protein production.

### Protein expression and purification

**Fig. 6:**
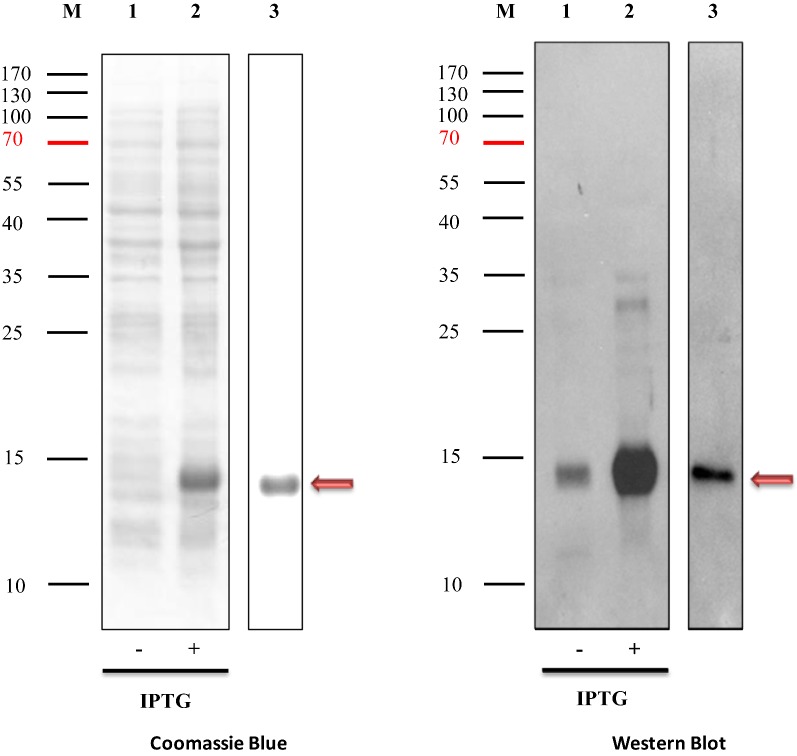
Expression and purification of recombinant cytosolic H8VNAR using pET-28a vector. Proteins were separated on 15% SDS-PAGE and stained with Coomassie Blue (left panel). H8VNAR was detected by Western Blot with anti-His tag antibody and a ~ 14 kDa band clearly apparent. Lane 1 represents total protein from non IPTG-induced bacteria cells; lane 2 represents total protein from IPTG-induced bacteria cells; and lane 3 represents purified cytosolic H8VNAR protein

**Fig. 7:**
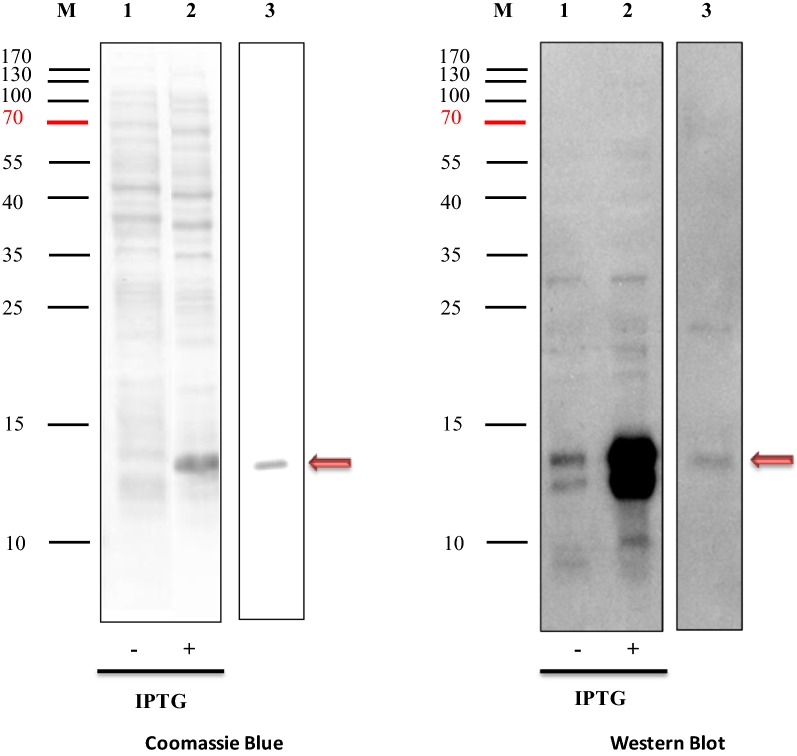
Expression and purification of recombinant periplasmic DsbA-H8VNAR using pDSB-28Y vector. Proteins were separated on 15% SDS-PAGE and stained with Coomassie blue (left panel). DsbA-H8VNAR was detected by Western Blot with anti-His tag antibody and a ~ 14 kDa band clearly apparent. Lane 1 represents total protein from non IPTG-induced bacteria cells; lane 2 represents total protein from IPTG-induced bacteria cells; and lane 3 represents purified periplasmic DsbA-H8VNAR protein

The expression and purification of H8VNAR using two different expression vectors yielded a clearly visible band on SDS-PAGE stained with Coomassie Blue (Figs. 6, 7). All H8VNAR proteins were present as well defined single bands at around 14 kDa in size, corresponding to the predicted molecular mass of H8VNAR including the 6× His tag (13.57 kDa). In Western Blot analysis, a “split” band was detected in the IPTG-Induced total cell lysates of *E. coli* processed for periplasmic expression (Fig. 7). However, this phenomenon was not observed in the cytoplasmic expression system (Fig. 6). Nevertheless, only a single band was detected in both H8VNAR proteins after purification, suggesting the soluble of recombinant V_NAR_ proteins can be obtained using bacterial expression system. No leakage of proteins from expression hosts could be observed. However, prolonged expression can lead to protein aggregation that eventually resulted in the formation of inclusion body in the *E. coli* expression host.

### Binding efficiency by sandwich ELISA

**Fig. 8:**
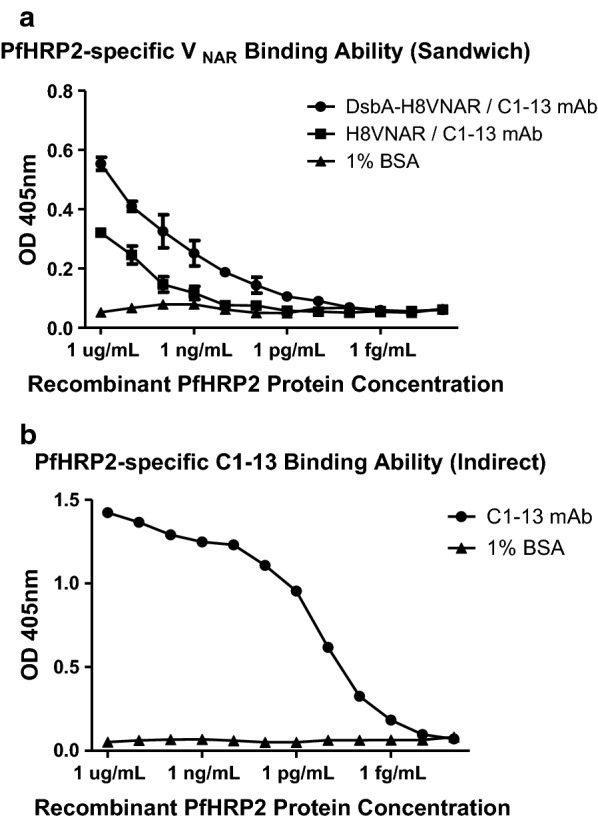
Comparison of binding efficiency of recombinant H8VNAR proteins with commercial mouse mAb C1–13 against tenfold serial diluted HRP2 proteins by ELISA. **a** The binding reactivity of purified cytosolic and periplasmic H8VNAR proteins was performed in a sandwich ELISA format. **b** The binding reactivity of positive control mAb C1–13 was performed in an indirect ELISA format. 1% BSA was used as negative antigen for rPfHRP2

The binding ability of recombinant V_NAR_ proteins was determined on ELISA plates in a sandwich format. The binding efficiency was compared with commercial mAb C1–13 that performed in an indirect ELISA format. From the screening assay, both V_NAR_ proteins gave positive responses when read on plate readers at OD 450 nm. The recombinant H8VNAR protein purified from periplasmic space showed better performance than that of cytosolic recombinant H8VNAR in terms of absorbance (Fig. 8a). At a concentration of rPfHRP2 of 1 µg/mL, the OD reading of periplasmic V_NAR_ protein (~ 0.58) was about twofold higher than that of protein produced by cytoplasmic expression (~ 0.32). However, these reactivities of the V_NAR_ proteins are inferior to the reactivity of mAb C1–13, the positive control in this experiment (OD of ~ 1.4 at the same concentration of rPfHRP2) (Fig. 8b). All OD readings demonstrated decreasing signal proportionally with reducing concentrations of rHRP2. No specific binding to BSA was observed for both recombinant H8VNAR proteins or mAb C1–13.

### Detection sensitivity by dot blot assay

**Fig. 9:**
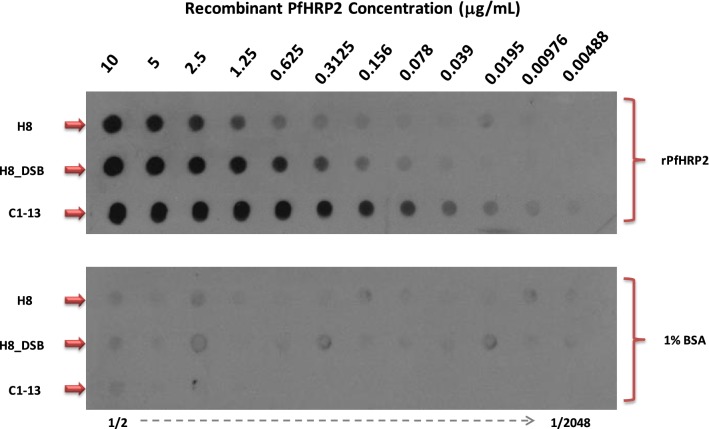
Comparison of binding reactivity of recombinant purified cytosolic and periplasmic H8VNAR against twofold serial diluted rPfHRP2 proteins in Dot Blot analysis. mAb C1–13 was a positive control. 1% BSA was a negative control

To further determine the detection sensitivity of recombinant antibodies, both cytosolic and periplasmic H8VNAR proteins were deployed as capture antibodies and examined in Dot Blot assay (Fig. 9). All recombinant antibodies reacted with rPfHRP2 protein showing different intensities similar to that observed in ELISA. Mouse mAb C1–13 showed the highest response with detection below 5 ng/mL. In contrast, the H8VNAR proteins exhibited lower binding affinity, even different binding reactivity was observed in both cytosolic and perisplamic produced H8VNAR proteins. The H8VNAR proteins produced in periplasmic space showed binding to the rPfHRP2 at a lowest concentration of 78 ng/mL. In comparison, the detection limit for the H8VNAR protein expressed in cytoplasm was 312 ng/mL. As expected, none of the recombinant antibodies tested reacted with 1% BSA, the negative control. Based on this observation, it was concluded that the sensitivity of periplasmic produced H8VNAR protein was superior to that of cytosolic protein, however both were less reactive than mAb C1–13. More Western Blot assays, designed to investigate possible cross reactivity to other malaria biomarker proteins for all of the selected V_NAR_ clones are planned. Further biological functions to investigate include reactivity for the native malaria protein, and thermostability will provide more insight in the diagnostic value of recombinant H8VNAR to rPfHRP2.

## Discussion

The small antigen binding domains of shark IgNAR (V_NAR_) have some unique properties in terms of thermostability and refolding capacity, which make them ideal antibody-based diagnostic tools. Hence, they have gained the interest of researchers focused on developing new binders for targeting antigens from various diseases. This study describes shark V_NAR_ antibodies that are specific against malaria antigens were successfully isolated and produced recombinantly in *E. coli*.

Following immunization of a wobbegong shark (*Orectolobus ornatus*) with three malaria biomarker proteins, the levels of IgNAR response against malaria recombinant PfLDH and PfHRP2 significantly increased in the wobbegong shark after the third immunization (Fig. 1), suggesting that the wobbegong shark could mount a humoral protective response to these antigens [35]. The fact that it took at least 3 boosts to observe an antibody response is likely due to sharks having a unique immune system where the antibody serum levels generally climb slowly compared to mammals. Therefore, it can take months until a significant antigen-specific titre is reached [36]. This phenomenon had also been indicated in work using hen egg lysozyme (HEL) to immunize nurse shark [25, 37–39]. This is presumably because pentameric IgM is responsible for providing early protection with high avidity interaction against invading pathogen in cartilaginous fish. In contrast, monomeric IgM likely IgG in mammals, and IgNAR are responsible for specific and high affinity binding response [35]. From these results, it is apparent that the immune system of the wobbegong shark studied herein was comparable to other shark families and appropriate IgNAR responses were identified.

After purification of mRNA from shark spleen, a V_NAR_ phage display library was successfully constructed using a T7 phage display system. Using this system, V_NAR_ displayed on the surface of T7 bacteriophage do not need to be extracted from the periplasmic layer of host cells which is a necessary step in M13 filamentous phage [40, 41]. Therefore, V_NAR_ fusion phage particles could directly be obtained through cell lysis. The functional phage titer yielded in this V_NAR_ domains primary library was estimated to have 5.3 × 10^5^ independent clones which is slightly lower than that of the libraries derived from naïve [28] and immunized [25] shark using M13 phage display system, and synthetic sharks using T7 phage display system [39]. Therefore, it was assumed that the library size may partly be influenced by factors such as packaging efficiency and phage display system (M13 or T7). In contrast to VH and VL genes in conventional antibody libraries, the genes encoding V_NAR_ repertoires were easier to clone [25, 41] with only two pairs of primers required to access the diversified shark immunoglobulin genes by PCR amplification of the V_NAR_ fragments from the spleen and PBL of immunized shark.

Sequence analysis indicates that about 90% of the clones in this library belonged to type 2 NAR. These data are in agreement with other V_NAR_ libraries derived from wobbegong sharks, indicating that the genes encoding IgNAR in the wobbegong family are prone to produce type 2 NAR domain [31]. However, genes encoding type 1 NAR representatives are commonly confined to nurse sharks [25]. In addition, about 27% of the clones in this library were found to possess three Cys residues in their CDR3 region. Although this number has rarely been described in IgNAR, Diaz et al. have previously reported it was found in some hypermutated clones in a nurse shark IgNAR [42].

In comparison with the human VH gene family, a higher degree of divergence in framework and hypervariable regions is exhibited in shark V_NAR_. First, the hypervariable regions of V_NAR_ are on average longer than those in VH. The average CDR3 length in conventional human and mouse VH is 12 and nine amino acids, respectively [43], whereas the length of shark V_NAR_ observed in this study was about 16–18 amino acids and in an agreement with those reported previously [25, 31, 42]. The hypervariability detected within CDR3 in V_NAR_ against malaria antigens after repetitive rounds of panning, suggests that these residues are contacting the antigen and that the somatic hypermutations in this region will take place during the affinity maturation [14, 32]. Furthermore, the enrichment of disulfide bridge in the long CDR3 region may cause the formation of disulfide loops that resulted to type 1 or type 2 V_NAR_ proteins configuration [44]. Therefore, to examine the quality of the V_NAR_ domain library constructed, the diversity in the CDR3 region, the different isotypes, and the position of the conserved canonical Cys residues were analysed and compared with other V_NAR_ domains that published in protein database.

Four rounds of biopanning resulted in the enrichment of antigen-specific phage antibodies directed against rPfHRP2, rPfLDH, and rPvAldolase proteins (Fig. 2). Interestingly, the pattern of enrichment was similar to the end titer of polyclonal IgNAR responses in vivo where the highest titers was rPfLDH-specific, rPfHRP2-specific, and followed by rPvAldolase-specific V_NAR_ clones. From this point of view, it is postulated the immunization process is an important factor to generate the high affinity single domain clones [25, 45]. In contrast to “naïve” antibody libraries, lesser rounds of panning cycles were needed to produce a highly specific signal which is in agreement with that reported from other immune phage display libraries [46]. Thus, the rapid selection of specific V_NAR_-displayed phage suggests that antigen-specific clones are well represented within the V_NAR_ population. The use of the T7 phage system has an advantage over M13 filamentous phage. In this system, phage amplification takes shorter time, and cell lysis can be observed by a visible reduction in OD of the culture after incubation.

The sequencing alignment revealed that all 26 clones specific to a malaria antigen protein were in-frame. Two distinct clones were identified targeting the recombinant rPfHRP2, whereas twelve clones each to targeting rPfLDH and rPvAldolase proteins. The amino acid residues found in the CDR3 regions differed for each antigen confirming that the CDR3 region is a major determining factor for the antigen binding specificity [47]. Moreover, the extraordinary length of CDR3 in shark V_NAR_ has been exhibited potentially penetrating into the cavities of antigen [48]. All of the selected V_NAR_ clones, except P9 and A9–3, appeared to contain at least one Cys residue in the CDR1 and CDR3 regions, suggesting that these clones belong to type 2 NAR family and are proposed to form an interloop of disulfide bond [49]. The length of the selected clones 10 to 24 amino acid residues with a mean of 17 amino acids (Additional file 2), indicating that longer hypervariable regions of the V_NAR_ could aid enlarging the actual surface of the antigen binding site, and that this might compensate for the absence of the antigen-binding surface area provided by the VL domain in conventional Fv of IgG [14, 31, 42].

Despite most of the selected clones demonstrating a positive response to corresponding antigen in monoclonal ELISA screening, it was difficult to differentiate the genuine clones from “sticky” clones using the phage display system. This phenomenon is observed quite often with phage display [50, 51]. Indeed, selection of highly cross reactive clones could possibly lead to production of recombinant V_NARs_ with inferior binding affinity. Another limitation observed with this T7 phage display system was the difficulty in isolating high affinity binders that contained disulfide bonds. The T7 phage display system is therefore more suitable for use in peptide discovery rather than antibody surface display [52, 53]. The polypeptides produced by T7 phage display system are synthesized in the reducing cytoplasmic environment of the host *E. coli*, which differs from phage secretion via the periplasmic space, as occurs in the filamentous M13 phage system. As a consequence, the proteins displayed on the surface of T7 phage may not be properly folded and therefore not ideally suited for functional screening for high affinity V_NAR_ clones. Because misfolded V_NAR_ presented on the surface of the T7 capsid could lead to false-positive plaques, additional rounds of competitive biopanning were undertaken to guarantee the specificity of targeted clones. In addition, the subsequent production of recombinant V_NAR_ proteins was performed in an alternate expression system.

H8VNAR was one of the highest affinity clones, and also the most frequently isolated during panning. In this clone, seven Cys residues were observed and predicted to form three disulfide bridges in its molecular structure. This prediction was done by DiANNA 1.1 Web Server (http://clavius.bc.edu/~clotelab/DiANNA/), a bioinformatics tool which is available online. Formation of disulfide bonds is known to be problematic in bacterial expression systems for eukaryotic proteins, particularly antibody fragments [54]. To achieve optimal folding, the production of proteins containing disulfide bonds needs to be manipulated in an oxidative environment of *E. coli* cells.

To make this possible, a signal sequence DsbA peptide was added to express PfHRP2-specific H8VNAR protein in *E. coli*. The recombinant H8VNAR expressed in periplasmic and cytoplasmic was then examined for their yield and binding activity. A periplasmic protein with an expected band at ~ 14 kDa was visible on SDS-PAGE (Fig. 7), suggesting that adding the DsbA signal sequence has efficiently directed V_NAR_ protein from cytoplasmic into the periplasmic space via the co-translational SRP pathway [55]. Thus, crystallography is planned to determine the conformation of recombinant H8VNAR and verify the presence of disulfide bond in this protein molecule.

When comparing the cytosolic H8VNAR and the periplasmic H8VNAR (or DsbA-H8VNAR), the binding capacity of DsbA-H8VNAR to recombinant PfHRP2 protein in Dot Blot assays was shown superior to that of cytosolic H8VNAR protein (Fig. 9). This insight indicated that periplasmic expression may potentially lead disulfide bond formation and proper folding of the protein thereby increasing the reactivity of recombinant antibody against the antigen epitopes [56, 57].

## Conclusion

In this work, the ability to raise the specific IgNAR from shark against malaria biomarker proteins by immunization was demonstrated. Meanwhile, a phage display V_NAR_ library was also successfully constructed by overlap extension PCR. Selection from this library resulted in several unique clones with specificity for each of three malaria antigens, suggesting the enrichment of affinity clones against malaria antigen proteins has been achieved by iterative biopanning and subsequent competitive biopanning. Also, the utilization of the DsbA signal peptide caused expression of soluble recombinant shark V_NAR_ proteins by enhancing folding capability was evidenced in the screening assays. Despite further optimization conditions under investigation, these findings and insights imply the expression of shark V_NAR_ is possible using the system designated in this study. The binding specificity and sensitivity of recombinant H8VNAR to the rPfHRP2 protein was demonstrated in a sandwich ELISA and a Dot Blot assay, respectively. Despite of that, further engineering including mutagenesis is planned in respect to improve the biological activity of H8VNAR towards malaria biomarker protein. Currently, a new semi-synthetic shark V_NAR_ library using M13 phagemids is being constructed whereby the performance of new clones against malaria antigens will then be presented.

## Additional files

**Additional file 1.** PCR amplification of randomly selected plaques from the V_NAR_ domains primary library using T7SelectUP (For) and T7SelectDOWN (Rev) sequencing primers. Lane M represents 100 bp ladder; lane 1–20 represents insert of single plaque; lane −ve represents negative control (PCR product with no cDNA template).

**Additional file 2.** Deduced amino acid sequences in hypervariable regions of V_NAR_ clones targeting to three malaria biomarkers. The non-canonical cysteine residue is highlighted in red colour. This table also indicates the length of CDR3, number of cysteine residue, and type of IgNAR family for each clone.

## Abbreviations

Aldolase: fructose 1,6-biphosphate aldolase
CDRs: complementarity determining regions
HRP: horseradish peroxidase
IgG: immunoglobulin G
IgNAR: immunoglobulin new antigen receptor
mAb: monoclonal antibody
PfHRP2: *P. falciparum* histidine-rich protein 2
pLDH: *Plasmodium* lactate dehydrogenase
RDTs: rapid diagnostic tests
V_NAR_: variable domain of new antigen receptor
