# Supplementary figures and images for: Isolation and characterization of malaria PfHRP2 specific VNAR antibody fragments from immunized shark phage display library
Source: Malar J. 2018 Oct 24;17:383. doi: 10.1186/s12936-018-2531-y (PMC6201582; doi:10.1186/s12936-018-2531-y)

Additional file 1:

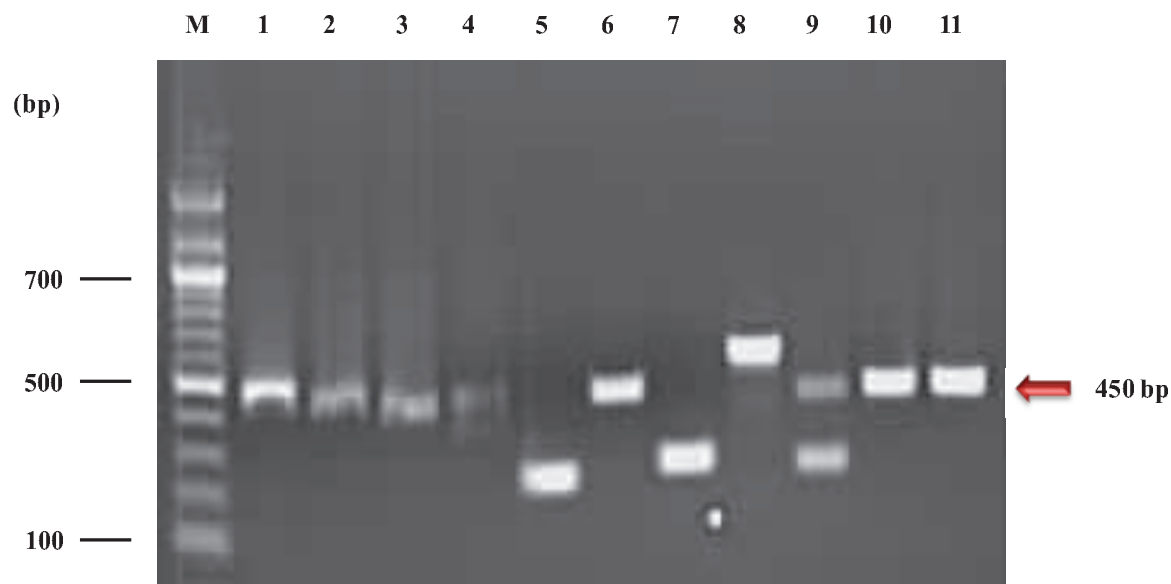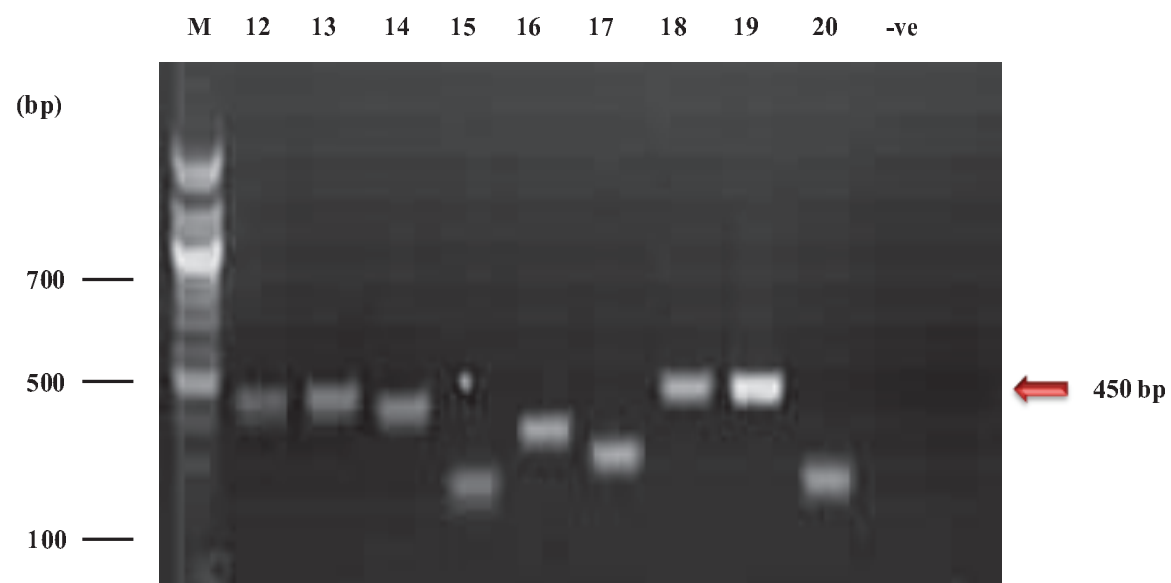

Supplement: Supplementary file 1 — Additional file 1. PCR amplification of randomly selected plaques from the VNAR domains primary library using T7SelectUP (For) and T7SelectDOWN (Rev) sequencing primers. Lane M represents 100 bp ladder; lane 1–20 represents insert of single plaque; lane −ve represents negative control (PCR product with no cDNA template). [file 12936_2018_2531_MOESM1_ESM.pdf]
