# Supplementary material for: Isolation and characterization of malaria PfHRP2 specific VNAR antibody fragments from immunized shark phage display library
Source: Malar J. 2018 Oct 24;17:383. doi: 10.1186/s12936-018-2531-y (PMC6201582; doi:10.1186/s12936-018-2531-y)
Supplement: Supplementary file 2 — Additional file 2. Deduced amino acid sequences in hypervariable regions of VNAR clones targeting to three malaria biomarkers. The non-canonical cysteine residue is highlighted in red colour. This table also indicates the length of CDR3, number of cysteine residue, and type of IgNAR family for each clone. [file 12936_2018_2531_MOESM2_ESM.pdf]

Additional file 2:

Clone	CDR1	HV2	CDR3	CDR3 length and number of cysteine	Type
**Anti-rPfHRP2**					
H8	DSANEFCR	TKEERISIS	EVCVCPCSQSEPDCKPGVIE	20 - 4	2
H18	DAGCGLYS	TDEQAISIG	EVCDFDRCDNCLSTWSRAYYE	21 - 3	2
**Anti-rPfLDH**					
P9	DTIYGLYS	TNEQRISIG	GAEASIWRYNY	11 - 0	2
P13	DSSKGLCS	TNGQRISIG	KAFDYDDAESCWHATHE	17 - 1	2
P16	DTKHPLCN	TNEQRISIG	KANVVRIATMRYCALEENY	19 - 1	2
P25	DGDCGLYR	TVEQTIPIV	GADAPNSCYVDGAGVTYY	18 - 1	2
P2-3	DASYSLCS	TNEETISIG	EACDALLCAAMKGLRCV	17 - 3	2
P3-3	DTIHEFCN	TNEERIEIG	ETCETYADYCCWGDYQ	16 - 3	2
P5-3	DTSYGLCS	TNEQRISIG	EACDDLCYGDYCGEWSGYVE	20 - 3	2
P6-3	HTEGSPCN	TNAARISIA	GVDDGGYTHADSVCGHWSEGY Y	22 - 1	2
P7-3	DTSYGLCN	TNEQRISIG	EACDAYSDCALCLSWRP	17 - 3	2
P8-3	DSNYGLLR	TNEQRISIG	ETSPYCDGWSRHGE	14 - 1	2
P9-3	DTTYGLCS	TNEQRISIG	GADDDVPGGSASCYGGPWDDR YN	23 - 1	2
P10-3	DTRYDPCN	TNQERISIG	ETSPLCDDWSPNGD	14 - 1	2
**Anti-rPvAldolase**					
A8	DASYGLCS	TNEQTISIG	QAYDGNSYSDCYWTTGDGY	19 - 1	2
A15	DSMHGLCS	TNEQRISIG	EQYMPTSCAAWSGFE	15 - 1	2
A16	DAKCALYR	TNEETISIG	EASTLARSHCDELELELGE	19 - 1	2
A17	DGRCGLHD	TNEQTISIG	NAYDWSQDASCYGSINYE	18 - 1	2
A1-3	DARCGLYS	TNEQRISIG	EACVRYSPKATAIGGVGYE	19 - 1	2
A3-3	DVVYGMCT	TNELGISIG	ETSPICDGWSPHGE	14 - 1	2
A4-3	DPGCGLYS	TGEQTISIG	GVDDGRSDDGRTFACYFAAG	20 - 1	2
A5-3	DTTYGLCS	TNEQGISIG	ETSPMCDGWSPNGE	14 - 1	2
A7-3	DTSSGLCG	TSEETIYNG	GGDSATISTGCRGVISL	17 - 1	2
A8-3	DVVYGMCT	TNELGISIG	EACDGGIYSDCYCAGDRAYFE	21 - 3	2
A9-3	DTSFGLYN	TNEQTISIG	KAYAEPGYRCSWNWE	15 - 1	2
A10-3	DTKHGLCS	TNEQRISIG	ETSPYCDGWSPNGD	14 - 1	2
